# Oral Solitary Fibrous Tumor: A Retrospective Clinico-Pathological Study and Long-Term Follow-Up

**DOI:** 10.3390/medicina57020152

**Published:** 2021-02-08

**Authors:** Tom Shmuly, Yehonatan Ben Zvi, Gabriel Chaushu, Ilana Kaplan

**Affiliations:** 1 Oral and Maxillofacial Surgery Department, Kaplan Medical Center, Rehovot 7642001, Israel; 2 Oral and Maxillofacial Surgery Department, Goldschleger School of Dental Medicine, Tel-Aviv University, Tel-Aviv 6934206, Israel; gabi.chaushu@gmail.com; 3 Oral and Maxillofacial Surgery Department, Rabin Medical Center, Petah-Tikva 4941492, Israel; yonident@gmail.com; 4 Pathology Department, Rabin Medical Center, Petah-Tikva 4941492, Israel; dr.ilanakaplan@gmail.com; 5 Oral Pathology Department, Goldschleger School of Dental Medicine, Tel-Aviv University, Tel-Aviv 6934206, Israel; 6 Pathology Department, Sackler School of Medicine, Tel-Aviv University, Tel-Aviv 6934206, Israel

**Keywords:** solitary fibrous tumor, hemangiopericytoma, oral cavity, CD34, immunohistochemistry

## Abstract

*Background and Objectives:* This was a retrospective single-center study to analyze and describe the clinical and histological features of all cases of oral solitary fibrous tumor (SFT). Study design: the study included all consecutive cases of oral SFT diagnosed between 2008–2018 at a single tertiary center. *Materials and Methods:* Clinical data was retrieved from medical charts. The diagnosis of oral SFT was based upon the morphologic features of the lesions, in routine hematoxylin and eosin (H&E) stained sections and confirmed by immunohistochemical analyses including CD34, CD99, Bcl2, and stains for STAT6. *Results:* Seven cases of oral SFT were found. Of these, three (42%) were in males and four (58%) in females. The age range was 24–63 years (mean 47 ± 13). Four (58%) lesions were located in the buccal mucosa, two (28%) in the labial mucosa and one (14%) on the floor of the mouth. The diameter ranged between 3–50 mm (mean 22 ± 14 mm). All patients were treated with local excision. Follow-up periods were between 2–74 months (mean 41 ± 27). No recurrences were reported. *Conclusions:* We present a series of oral SFT, which were all non-aggressive in presentation and did not recur after conservative surgery (local excision) over a relatively long follow-up period.

## 1. Introduction

Solitary fibrous tumor (SFT), is a fibroblastic mesenchymal tumor that was first described by Klemperer and Rabin in 1931 as a distinct pleural entity, which was hypothesized to be of submesothelial origin [1]. These tumors are pluripotent and can differentiate to mesothelial cells, fibroblastic cells, or other cell types [2]. In recent years this tumor has been documented in almost every anatomic location. In the head and neck region, SFT may be found in the orbit, nose and paranasal sinuses, major salivary glands, larynx, thyroid, and the deep soft tissues of the neck, among other places [3,4,5,6,7,8]. Oral SFT is a rare entity—only 150 cases were reported until May 2019, and most were case reports with only a few case series. Out of the 150 oral SFT reported cases, only 14 (10%) were at the time classified as malignant [9]. In the latest edition of the WHO Classification of Tumors [10], the SFT’s classification as benign or malignant has been excluded, instead a set of criteria to predict the risk of metastasis has been included. Histologically, SFT reveals a collagenized stroma containing benign-looking, spindle-shaped tumor cells, with occasional nuclei showing mild nuclear atypia. Focal areas of dense hyalinization and characteristic staghorn-shaped blood vessels are consistently present. Immunoreactivity for CD34, Bcl2, and CD99 is helpful to confirm the diagnosis [11]. Furthermore, a recurrent chromosomal fusion affecting NAB2 and STAT6 genes was described in SFT, leading to a consistent nuclear expression of STAT6 protein, making STAT6 a sensitive and specific marker for SFT [12].

Alawi et al. concluded that clinical behavior of SFT, in general, remains unpredictable, and suggested a long-term follow-up of all patients with SFT [13]. The purpose of the present study is to describe the clinical and histological features of all cases of oral SFT diagnosed and treated in Rabin Medical Center in Petach-Tikva, Israel during the years 2008–2018.

## 2. Materials and Methods

The study is a retrospective analysis, which included all consecutive cases of oral SFT diagnosed between 2008–2018 at a single tertiary center (Rabin Medical Center, Petach-Tikva, Israel). All cases of confirmed SFT were retrieved from the archives of pathology. Patient charts were reviewed and clinical data, including age, sex, clinical presentation, duration of symptoms, tumor location, tumor size, extent of surgery, and follow-up were collected.

All cases with a diagnosis of oral SFT were reviewed, all lesions were examined by an oral pathologist (IK) to confirm the pathological diagnosis. The diagnosis of oral SFT was initially based upon morphologic features of the lesions, in routine hematoxylin and eosin (H&E) stained sections. All initial diagnoses were confirmed by immunohistochemical analyses and stains for STAT6 were added in the older cases diagnosed prior to the availability of this antibody.

For this, 3-mm thick sections mounted on silane-coated glass slides were deparaffinized and rehydrated in graded ethanol solutions. Immunohistochemical staining was done using monoclonal antibodies for CD34 (clone QBEnd/10—cell marque), CD99 (clone EPR3097Y, cell marque—1:100), Bcl2 (E17 cell marque 1:25) and STAT6 (clone EP325—BioSB—1:25).

This study was approved by the institutional Ethics Committee (0563-19-RMC, 7.10.2019).

## 3. Results

A search of the archives of the pathology department for all cases diagnosed as SFT (2008–2018) yielded a total 45 cases; 17 of these were from the head and neck region, of which only 7 were from the oral cavity. Out of the 17 head and neck cases, 10 were, at the time, diagnosed as malignant and 7 were, at the time, diagnosed as benign. In this retrospective study we chose to focus only on the seven oral cases.

**Figure 1:**
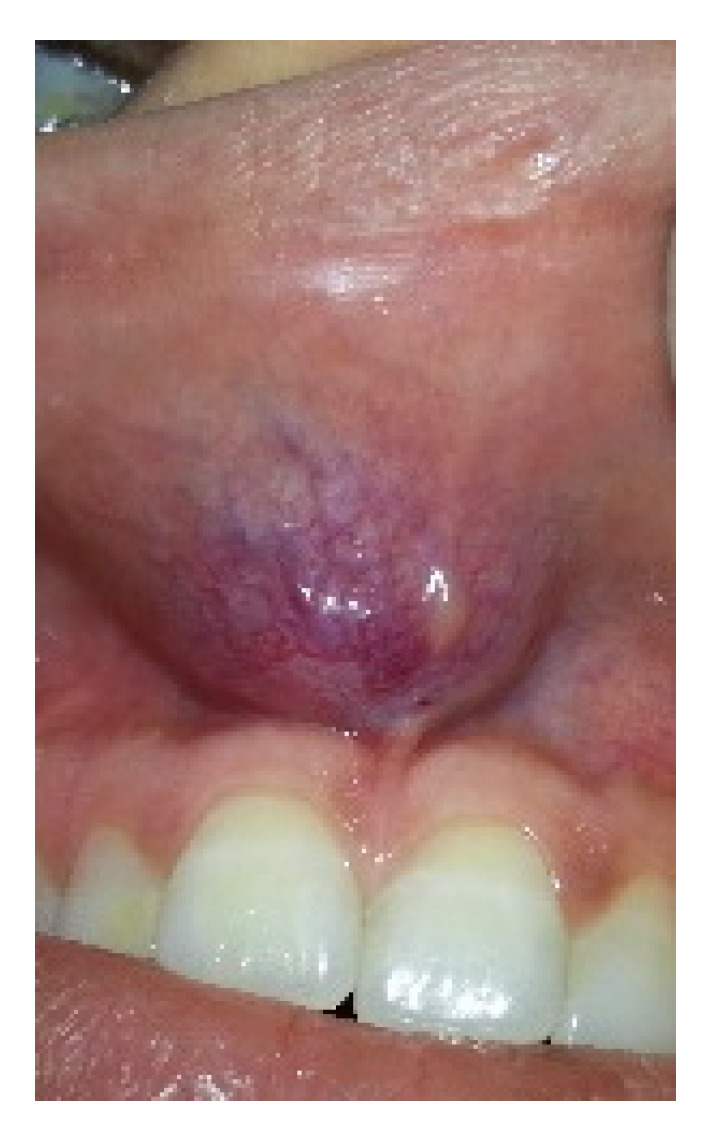
Shows a solitary fibrous tumor (SFT) as a subepithelial lesion in the labial mucosa. Note that it can’t be clinically distinguished from numerous other subepithelial oral lesions.

**Figure 2:**
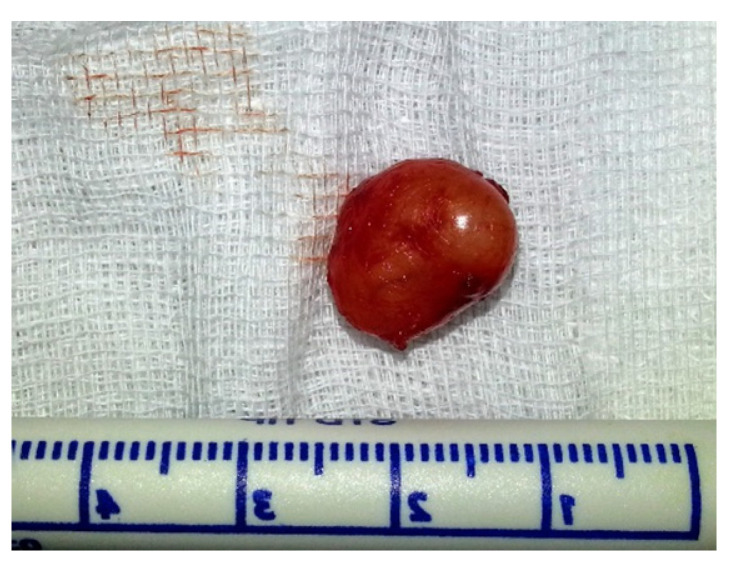
Shows a macroscopic view of the lesion after excision. Note the well-defined mass with a firm and rubbery consistency.

From a total of seven patients with oral SFT, three (42%) were males and four (58%) females. The age range was 24–63 years (mean 47 ± 13). Four (58%) lesions were located in the buccal mucosa, two (28%) in the labial mucosa and one (14%) on the floor of the mouth. The diameter ranged between 3–50 mm (mean 22 ± 14 mm). In at least two patients’ records, the lesion was described in the area of the Linea Alba, and local trauma was suggested as the cause. All patients were treated with local excision, of which six were performed under local anesthesia in the outpatient clinic and one under general anesthesia and included vascular embolization and local excision. Table 1 shows patients’ details including location, surgical procedure, and follow-up periods. Figure 1 shows an example of a subepithelial lesion in the labial mucosa, later diagnosed as SFT. Gross examination of the resected specimens revealed well-defined masses with a firm and rubbery consistency, exhibiting a grayish smooth surface. Figure 2 shows a macroscopic view of the lesion after excision.

Routine microscopic examination in H&E-stained sections revealed a cellular tumor, with uniform and bland-looking spindle cells, without any organized pattern (patternless). The matrix was usually collagen-rich, and irregular blood vessels with a stag-horn morphology present within the tumor mass. All cases were well-circumscribed and non-infiltrative, only one case showed mild atypia, none exhibited necrosis, and mitotic activity was low in all cases, well below the threshold of 4 mitosis/10 high-power fields (HPF). Therefore, all of the cases in the present series conform with the criteria for low-risk for metastasis and recurrence [14,15].

**Figure 3:**
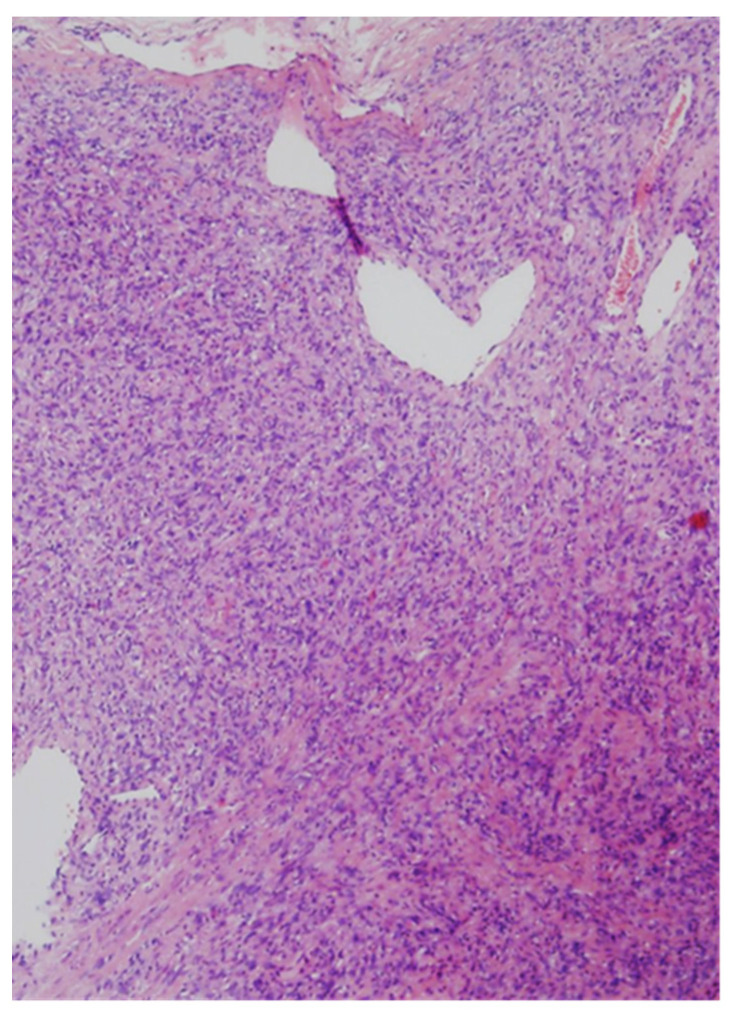
Low magnification showing a cellular spindle cell tumor with a “patternless pattern”. Irregular branching vessels are visible. (hematoxylin and eosin (H&E) stained, original magnification ×40).

**Figure 4:**
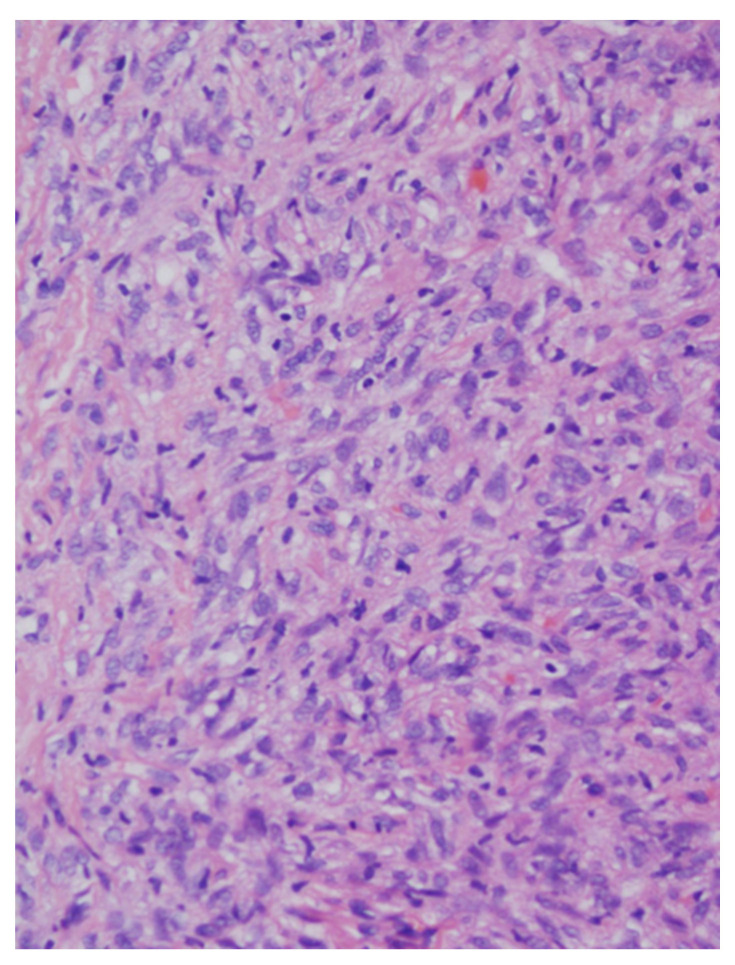
At a higher magnification the tumor cells are spindle-shaped, monomorphic, and bland-looking (H&E stained, original magnification ×200).

**Figure 5:**
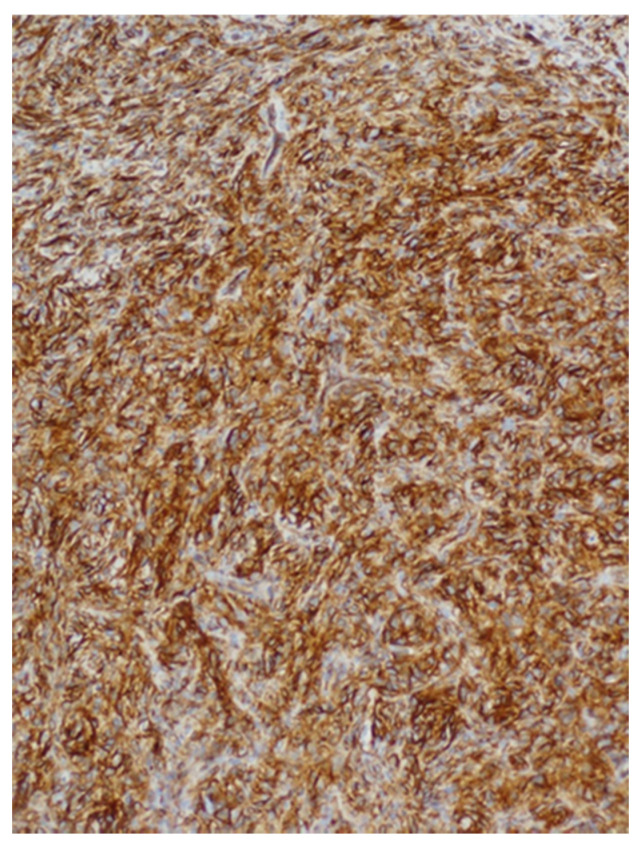
Immunohistochemical staining for CD34 shows intense and diffuse staining of most of the tumor cells.

**Figure 6:**
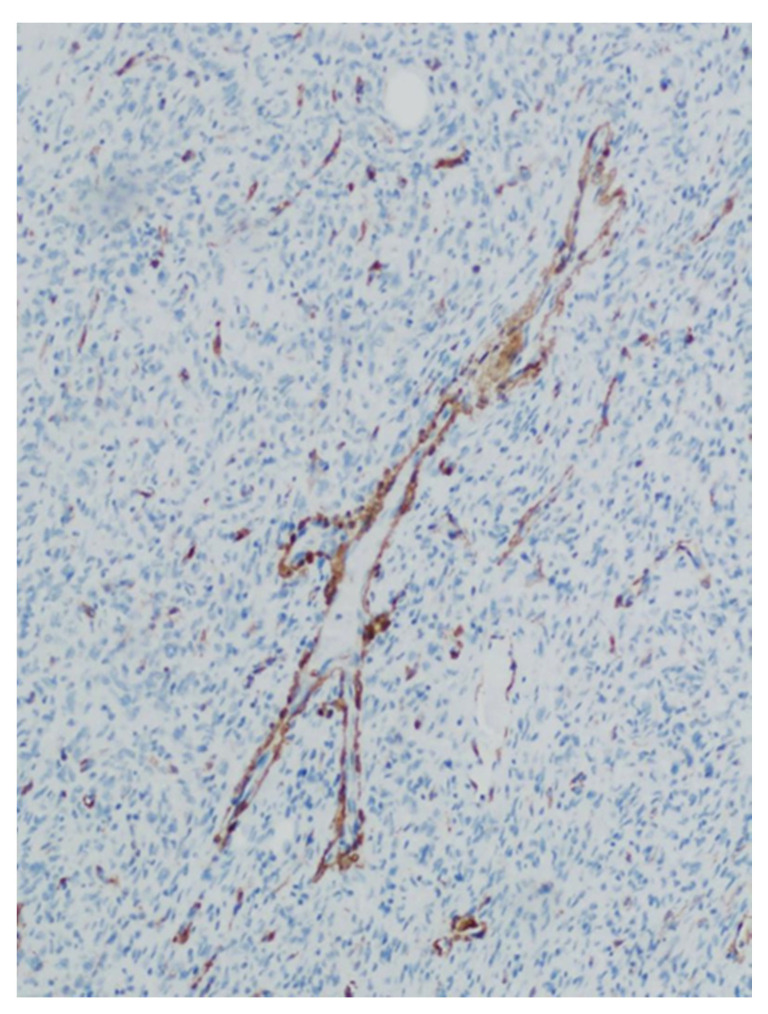
CD31 stains only the endothelial cells in the vessels, but it is negative in the remaining tumor cells. The particular branching vascular pattern is demonstrated in this area.

**Figure 7:**
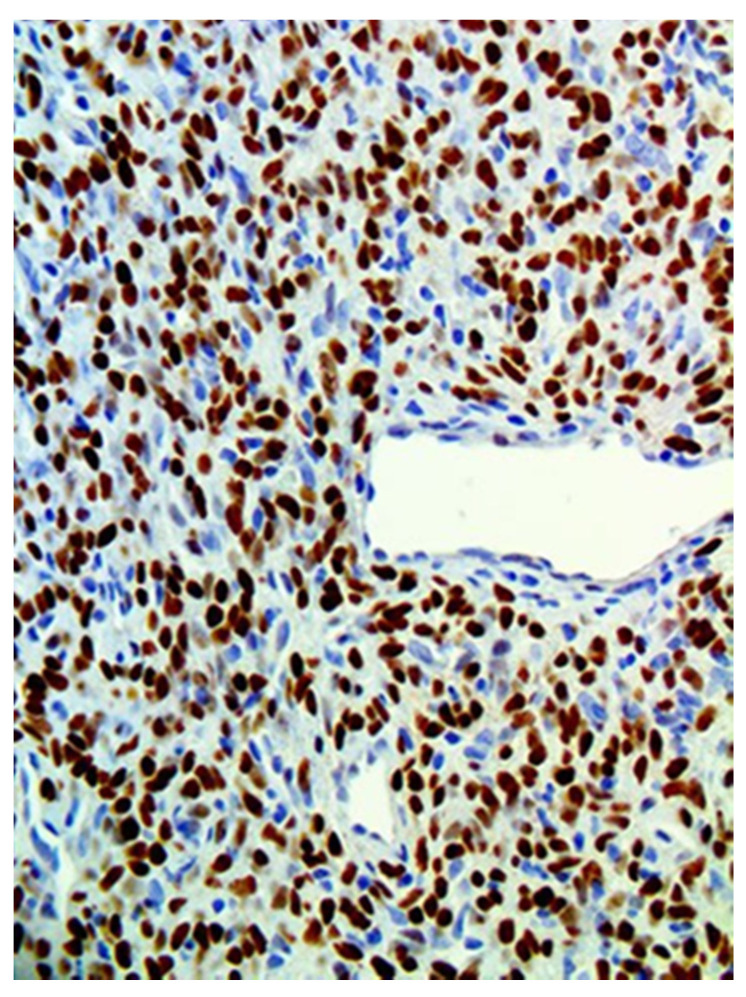
STAT6 stains strongly and diffusely the nuclei of the tumor cells, a pathognomonic finding in SFT.

In immunohistochemical analysis, tumor cells were stained for CD34, CD99, and Bcl2. All cases were also positive for STAT6 (Figure 3, Figure 4, Figure 5, Figure 6 and Figure 7). Table 2 shows immunohistochemical staining for each patient. None of the cases included showed any overt atypia or mitotic activity, suggesting a benign behavior of all reported cases in this series.

The follow-up period was between 2–74 months (mean 41 ± 27). One patient (patient number 5) died due to lymphoma. None of the cases recurred during follow-up.

## 4. Discussion

SFT of the oral cavity is an uncommon mesenchymal tumor [11]. In this study we described seven cases of oral SFT, of which all were treated with conservative local excision with a follow-up time of up to 74 months, with no recurrence reported. In the present study, SFT was diagnosed in a wide age group from young adults to elderly (24–63 years), an age variance consistent with previous publications [9,15,16].

A previous report suggested trauma may be an etiological factor in oral SFT [13]. Two cases in the present series also reported previous trauma to the area of the lesion, although this may not necessarily prove a cause-and-effect relationship. In previous reports, the buccal mucosa was the most affected site [9,11,16]. This fact was confirmed in the present series as well, with four of the seven cases occurring in the buccal mucosa. It should be noted that the buccal mucosa is also the most frequent area in the oral cavity to suffer from local trauma [13]. Local trauma my cause inflammation in the oral cavity which may be influenced by also other factors. Recent studies by Isola et al., have demonstrated the importance of nutraceutical agents on the healing processes and inflammatory reactions in the oral cavity, and also of a possible association between chronic cardiovascular disease and inflammationin the oral cavity [17,18].

A set of criteria to predict the risk of metastasis and recurrence was introduced by Salas et al., and adopted in the fifth edition of the WHO Classification of Tumors. Rather than the designation of benign or malignant, these features include extrapulmonary location, hypercellularity, nuclear atypia, infiltrative borders necrosis, and mitotic count higher than 4/10 HPF [10,14]. During the present study period, a total of 17 head and neck cases were diagnosed in our institution (including the 7 oral cases represented in this study), of which 10 can be classified as carrying a potential risk for metastasis and recurrence by the aforementioned criteria: all 10 cases showed hypercellularity, 5 out of 10 cases showed nuclear atypia, 2 cases showed necrosis, and a mitotic count of 4/10 HPF or higher was observed in 3 cases; resulting in 1 case being classified as grade 1, 7 as grade 2, 1 as grade 3, and 1 classified as 2–3. Nunes et al., in his literature review (published in early 2020) of SFT in the oral cavity reports that out of 150 cases published 136 (90.7%) were benign and 14 (9.3%) were malignant [9]. Also out of 153 head and neck cases reported in a literature review by Cox et. al. in 2010, only 10 (6.5%) showed malignant features [19]. Our findings represent a higher frequency of malignancy/high risk than reported before from other countries. Whether these differences represent ethnic variability can only be speculated from the existing data. All seven oral SFT cases in our study were low-risk, and exhibited none of the features predicting metastasis or recurrence in the recent classification. Thus, from the present cases and the literature it can be concluded that oral SFT are predominantly of the low risk for metastasis variant.

SFT exhibits non-specific clinical manifestation, and would be in the differential diagnosis together with other soft-tissue submucosal lesions (such as neurofibroma, schwannoma, myofibroma, leiomyoma, and salivary gland neoplasms), making the clinical recognition of oral SFT very unlikely [9,11]. Final diagnosis is based on histopathological features and confirmed by immunohistochemistry. Microscopic characteristics, which should raise the possibility of SFT, include circumscribed spindle cell proliferation with a variably vascular and collagenized stroma. Finding staghorn-shaped vessels in the lesion is a helpful finding, and the immunostains that can finalize the diagnosis include positive staining for CD34, Bcl2, CD99 [16,20,21]. Recently, staining for STAT6 was shown as a sensitive and specific marker for SFT [12].

In this case series, all reported lesions were treated with conservative local excision and no recurrence was reported with a relatively long follow-up period (2–74 months). Our results are consistent with those of Nunes et al. In his review of the literature of 150 cases, 97.3% of lesions were treated conservatively, 0.7% with resection and 2% with resection and radiotherapy, also, after a mean follow-up period of 24 months a recurrence rate of only 0.7% was reported [9]. In contrast, when treating SFT in general, and not in the oral cavity, an en-bloc resection with negative margins is advised. There is little evidence that adjuvant chemotherapy and radiation following complete surgical resection is beneficial, and thus they are not routinely performed [22,23]. Demicco et al. reports 110 cases of total-body SFTs. Of these, 103 cases were treated surgically, out of which 15% patients also received adjuvant therapy (radiotherapy and/or chemotherapy) and after a median follow-up period of 48 months, a large number of patients—29%—had local recurrence and or metastasis [24].

## 5. Conclusions

We present a series of oral SFTs, which were all non-aggressive in presentation and did not recur after conservative surgery (local excision) over a relatively long follow-up period. Oral SFT is thus a rare tumor in the oral cavity, yet it is non-aggressive, with low risk for metastasis in the vast majority of cases.

## Figures and Tables

**Table 1:** Summary of clinical features of SFT in the present series.

Patients	Age (years)	Sex	Location	Size (mm)	Surgical Procedure	Follow-Up (months)	Recurrence
1	43	M	Buccal mucosa	25	Local excision	30	No
2	24	F	Upper labial mucosa	14	Local excision	2	No
3	41	F	Buccal mucosa	17	Local excision	60	No
4	60	M	Floor of mouth	35	Local excision	74	No
5	63	M	Lower labial mucosa	3	Local excision	NA	NA
6	60	F	Buccal mucosa	50	Local excision+ Embolization	66	No
7	43	F	Buccal mucosa	14	Local excision	15	No

NA—not available.

**Table 2:** Summary of the histological features of oral SFT in the present series.

Patients	CD34	CD99	STAT6	Bcl-2
1	+	+	+	NA
2	+	NA	+	NA
3	+	NA	+	+
4	+	NA	+	+
5	+	NA	+	NA
6	+	NA	+	+
7	+	NA	+	NA

NA—not available.

## Data Availability

The data will be available upon request from the corresponding author via Email.
